# Effects of intermittent theta burst stimulation add-on to dialectical behavioral therapy in borderline personality disorder: results of a randomized, sham-controlled pilot trial

**DOI:** 10.1007/s00406-024-01901-0

**Published:** 2024-09-19

**Authors:** Milenko Kujovic, Christian Bahr, Mathias Riesbeck, Daniel Benz, Lena Wingerter, Martina Deiß, Zsofia Margittai, Dirk Reinermann, Christian Plewnia, Eva Meisenzahl

**Affiliations:** 1 Department of Psychiatry and Psychotherapy, Medical Faculty and University Hospital Düsseldorf, Heinrich-Heine-University Düsseldorf, Düsseldorf, Germany; 2 Department of Psychiatry and Psychotherapy, University Hospital of Psychiatry and Psychotherapy, Tübingen, Germany

**Keywords:** Borderline Personality Disorder, Dialectical Behavior Therapy, rTMS, iTBS

## Abstract

Dialectical behavioral therapy (DBT) and repetitive transcranial magnetic stimulation (rTMS) are both effective in borderline personality disorder (BPD). We hypothesized that intermittent theta burst stimulation (iTBS), a modified rTMS protocol that provides unilateral stimulation to the left dorsolateral prefrontal cortex, would enhance the effects of DBT and reduce BPD-specific symptoms more than sham stimulation. We performed a single-blind, randomized, sham-controlled pilot study to evaluate iTBS as an add-on to 8-week DBT for BPD in routine inpatient treatment. A total of 53 BPD patients were randomly assigned to either iTBS (n = 25) or sham stimulation (n = 28) in weeks 4–8 of DBT; 40 patients were eligible for inclusion in the analyses according to pre-specified criteria (≥ 16 of 20 iTBS sessions). The primary endpoint was change on the 23-item Borderline Symptom List; secondary endpoints were changes in depressive symptoms and general level of functioning. A mixed model repeated measures analysis with a 2 × 2 factorial between-subjects design showed no significant effect of add-on iTBS treatment, but a distinct trend was observed in favor of iTBS (Cohen’s d = 0.23 for group difference). We found a main effect of DBT with and without iTBS over time, indicating efficacy of 8 weeks’ DBT (d = 0.89–1.12). iTBS may be beneficial as an add-on to DBT in the long term and warrants further evaluation in larger studies. *Trial registration* Registered at drks.de (no. DRKS00020413) on January 13, 2020.

**Supplementary Information:**

The online version contains supplementary material available at 10.1007/s00406-024-01901-0.

## Background

Borderline personality disorder (BPD), a severe mental illness characterized by persistent emotional instability, impulsivity, self-harming tendencies, distorted self-image, and impaired social functioning, causes significant distress and diminished quality of life [1, 2]. Studies have found prevalence rates of 0.7–4.5% [3] and up to 22% among inpatients [1, 4]. The lifetime prevalence is estimated at about 5%, and the disorder is associated with a broad range of comorbidities, particularly depression [5, 6].

In Germany, in- and outpatient care of BPD is considered as improvable due to lacking therapists with specific training, e.g., in dialectical behavioral therapy (DBT) [7]. Currently, the estimated ratio of certified therapists to BPD patients is 1:1102 [8]. Furthermore, the direct and indirect costs of BPD are estimated to be as high as €40,000 (per case and year). Accordingly, new approaches that could improve BPD health care are required.

Guidelines [9] recommend psychotherapy as the primary treatment in BPD. Some therapies, e.g., DBT, were developed to meet the specific needs of BPD symptoms [10]. According to the German BPD guideline [11], DBT has the strongest evidence level among the BPD treatments. Numerous randomized controlled trials, meta-analyses and systematic reviews have consistently demonstrated the efficacy of DBT in BPD [10, 12, 13]. Although routine inpatient DBT programs in Germany last 12 weeks [14], a shorter, 8-week approach was shown to be equally effective [15].

Research indicates that BPD involves neurobiological changes, e.g., in dysfunctional fronto-limbic brain networks [16–19], which are associated with reduced activity in the frontal regions and increased activation in the limbic system. This imbalance is thought to lead to reduced inhibitory control of emotion-eliciting brain regions, resulting in impaired emotion regulation [20].

Some studies suggest that DBT may affect neural activity [17] and could enhance neural plasticity in brain regions associated with emotional dysregulation, potentially reducing symptoms and improving overall functioning [21]. For example, a recent review [22] found significant reductions in amygdala and anterior cingulate cortex activity in BPD patients after DBT. Additionally, various studies reported that DBT decreased inferior frontal gyrus activation when patients were exposed to exciting stimuli and increased activation in response to inhibitory control exercises [22].

Repetitive transcranial magnetic stimulation (rTMS) has gained attention as a potential treatment for various psychiatric disorders [23]. Several meta-analyses have analyzed the evidence for the treatment of major depression [24, 25], but little evidence and few recommendations are available for other disorders [23]. Recently, systematic reviews focused on the application of rTMS in BPD [26–28] because of findings of reduced impulsivity [29] and general symptom reduction [30]. One review stated that rTMS could be well combined with other treatments, such as psychotherapy [26].

A commonly stimulated brain area in rTMS is the dorsolateral prefrontal cortex (DLPFC) [28, 31]. The DLPFC plays a crucial role in various cognitive processes, including working memory, creative thinking, focused attention, and decision-making, is associated with cognitive control, and emotion regulation [32], and may be related to mood modulation [33]. When the function of the left lateral prefrontal cortex was temporarily disrupted by rTMS in healthy individuals, impulsive behavior increased and self-control regulation decreased [34]. The left DLPFC is associated with regulation of impulsivity, risk-taking behaviors, and analytical processing, whereas the right DLPFC is associated with reflective tendencies, slower information processing, and avoidance of impulsive decisions [35]. The DLPFC is also involved in maintaining the balance between assessing the significance of emotions and regulating the valence of emotional experiences [32, 33, 36].

rTMS can be used to stimulate the left, right, or bilateral DLPFC [28, 31]. In depression, the left DLPFC is most commonly stimulated, and high-frequency rTMS is used [31, 37]. In BPD, patients show functional and structural abnormalities in the fronto-limbic network, which comprises the amygdala, insula, anterior cingulate cortex, orbitofrontal cortex, and DLPFC brain regions [38, 39]. Although brain stimulation studies show encouraging results [28], causal assumptions should be drawn cautiously [20].

The aim of BPD treatment is to mitigate impulsive behavior and promote increased top-down inhibitory control. Because both DBT and rTMS target the above-mentioned imbalance in BPD, combining DBT and rTMS may have beneficial effects in BPD [26].

A common approach for TMS-protocols is theta-burst stimulation (TBS), which has the advantages of having a short duration and using low-intensity stimulus pulses [40]. This is expected to result in higher patient acceptance and lower dropout rates. Originally, TBS aimed at imitating the theta rhythm, which occurs in mammals during exploratory behavior and memory processes [40]. TBS is also associated with induction of long-term potentiation and long-term depression [40, 41]. TBS protocols comprise intermittent TBS (iTBS) and continuous TBS (cTBS): The former increases the excitability of neurons, whereas the latter depresses it [23, 41, 42]. The common protocol for iTBS involves 10 bursts of 50 Hz triplets separated by 8-s non-stimulation intervals, making a total of 600 pulses over 190 s, and causes increased excitability in the motor cortex for up to 60 min [41, 42].

New approaches combine rTMS or iTBS with psychotherapeutic interventions because the combined interventions may potentially have better outcomes than individual ones [26]. For example, one review [43] suggested that noninvasive brain stimulation could enhance cognitive behavioral psychotherapy (CBT) for mood and anxiety disorders [43], and a study [44] showed that iTBS add-on to CBT could be beneficial regarding abstinence in smoking cessation.

Because both DBT and rTMS have shown efficacy for BPD-specific symptoms, it appears worthwhile to evaluate combination treatment. However, the shorter duration and lower intensity stimulus pulses of iTBS compared with rTMS are expected to be better tolerated by BPD patients and thus to be associated with better compliance. To our knowledge, so far no study has investigated a combination of DBT and iTBS in BPD. Therefore, we performed a randomized, single-blind, placebo-controlled pilot trial to investigate iTBS as an add-on to 8 weeks’ DBT in a routine psychiatric treatment setting [45]. We hypothesized that augmenting DBT with iTBS would result in greater reductions in BPD-specific symptoms compared with placebo/sham stimulation. Since comorbidity rates of BPD and depression are high (up to 80%, [6]) and rTMS has proven efficacy for depression treatment [24, 25] patients with both diagnosis (BPD and depression) have been included.

## Methods

### Procedures

Patients with BPD participating in an 8- to 12-week routine inpatient DBT on a specialized ward at the Department of Psychiatry, LVR Clinical Center Düsseldorf, Heinrich Heine University, Düsseldorf, Germany, were recruited within the first 4 weeks of DBT. After reaching informed consent patients were randomly allocated (1:1) to receive 20 sessions of iTBS (DBT + active iTBS group) or 20 sessions of sham stimulation (DBT + sham); active iTBS or sham stimulation was administered once per day from Monday to Friday in week 5 to 8 of DBT. Randomization was based on an algorithm created with the programming platform MATLAB. Patients were blind to the condition, but the study staff were not. The individuals who performed data analysis were blinded to group allocation.

The study protocol was completed in July 2019 and published on September 15, 2023 [45]. The trial was registered in the German Clinical Trials Register (DRKS) on January 13, 2020 (registration number DRKS00020413). All participants provided written informed consent by signing the declaration, patient information, and data protection documents. The study was approved by the ethics committee of the medical faculty at Heinrich-Heine-University, Duesseldorf, Germany, on 13 December 2019 (reference number: 2019–637; with 1st amendment 27 July 2020 and 2nd amendment 9 February 2021) and performed in compliance with all relevant laws and institutional guidelines and the Code of Ethics of the World Medical Association (Declaration of Helsinki).

Routine inpatient DBT was based on an adaptation of a program from Bohus et al. [46]. It followed a structured, modularized approach that included the modules *skills training*, *emotion regulation*, *interpersonal skills,* and *mindfulness* and comprised weekly individual and group therapy sessions. To maintain compliance with the DBT manual, comprehensive training in all modules was given to all staff members, including medical personnel, psychotherapists, nursing staff, occupational therapists, and others, by the Dachverband DBT e.V.; these training sessions were conducted by certified personnel affiliated with the DBT association. For further details, see Kujovic et al. [45].

### Inclusion/exclusion criteria

Patients were included if they were diagnosed with both BPD and comorbid major depression and had no other psychiatric comorbidities. Diagnoses were confirmed with the Diagnostic Interview for Mental Disorders (Mini-DIPS OA; [47]) and the Structured Clinical Interview for DSM-5 Personality Disorders (SCID-V-PD; [48]). Other inclusion criteria were age 18–45 years; sufficient knowledge of German and able to give written informed consent; in case of drug treatment, stable intake of therapeutic doses for two weeks before the start of the stimulation phase and during the 4-week stimulation phase; and, for female patients, negative pregnancy test and willingness to use contraception for the duration of the study. Exclusion criteria were a history of seizures (epilepsy), metallic foreign objects in the skull, presence of several/extensive tattoos in the head region, significant brain malformations or tumors, cerebrovascular events, traumatic brain injuries, neurodegenerative diseases, brain surgery, deep brain stimulation, other intracranial implants, cardiac pacemaker, other serious physical illness, other psychiatric comorbidities besides major depression and BPD, acute suicidal thoughts (Montgomery-Åsberg Depression Rating Scale [MADRS, [49]] score > 4 for question 10), tinnitus, pregnancy, claustrophobia, and current or previous treatment with electroconvulsive therapy or vagus nerve stimulation. Patients taking anti-epileptic medication, including benzodiazepines, at a dose equal to or greater than 1 mg/day of lorazepam, patients under legal guardianship with limited ability to give consent, and patients who had previously participated in DBT were excluded. As predefined in the study protocol, patients with more than four missed iTBS or sham sessions were excluded from the statistical analyses (see Kujovic et al. [45]).

### iTBS and sham treatment

For iTBS stimulation, we used a PowerMAG Research 100 magnetic stimulator [50] and a PMD70-pCool figure of eight coil that were located on the ward where the routine psychiatric treatment was provided. Before the first treatment and two weeks later, the resting motor threshold was automatically determined by electromyography, which involved integrating the motor evoked potential and applying an algorithmic approach to determine the threshold [51–53]. To locate the left DLPFC, we used the Beam-F3 method [54]. The stimulation intensity was 80% of the resting motor threshold. During each iTBS treatment session, a total of 600 stimuli were delivered. Furthermore, each treatment unit lasted three minutes and 12 s because the stimulation was applied intermittently and consisted of two seconds of stimulation followed by an eight-second pause. To ensure blinding of patients, for the sham condition we used a sham coil (PMD70-pCool-Sham, [55]) with a similar weight und sound as the iTBS coil; the sham coil generated a lower magnetic field strength, which enabled stimulation of the immediate scalp region without having an impact on the brain. Therefore, participants in the sham group experienced noises and physical sensations similar to those in the active group.

### Study endpoints

The primary outcome was the difference between the active and sham groups in the change in BPD symptoms from before to after the iTBS intervention, assessed with the 23-item Borderline Symptom List (BSL-23, [56]; see Supplementary Table S1 for timing). Secondary endpoints were the reduction in depressive symptoms as evaluated with Beck’s Depression Inventory (BDI-II, [57]) as a self-rating measure and the MADRS [49] as a clinician-rated measure. Furthermore, general functioning was assessed by trained clinicians with the Global Assessment of Functioning scale (GAF; [58]), and self-compassion was evaluated with Self-Compassion Scale (SCS; [59]). For a detailed description of the instruments, see Kujovic et al. [45].

### Sample size and power calculation

**Fig. 1:**
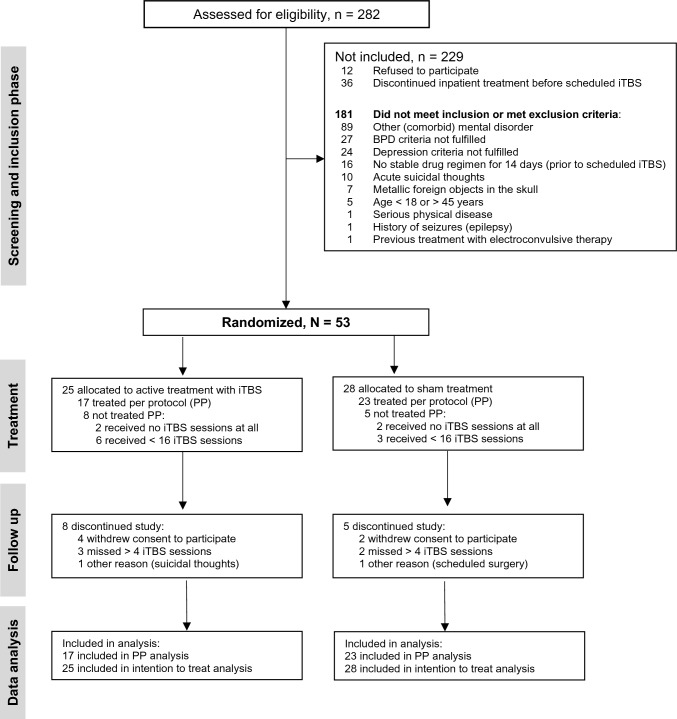
CONSORT flow diagram (according to Moher et al. [62]) of the screening and randomization process. iTBS, intermittent theta-burst-stimulation; PP, per protocol

Because limited research has been performed on non-invasive brain stimulation in BPD, few studies were available as a basis for the power analysis [26, 28]. However, two pilot studies [30, 60] indicated medium to large effect sizes for rTMS in BPD. Accordingly, a sample size of 40 was calculated to be sufficient to detect a small to medium effect with an alpha value of 0.05 and 80% power (for details see Kujovic et al. [45]). A total of N = 53 participants were included in the study, of which n = 40 were included in the data analysis (see Fig. 1); this drop-out rate of 24.5% corresponded to the expected dropout rate of 25%.

### Statistical analysis

We used a 2 × 2-factorial between-subjects design to evaluate differences in primary and secondary outcome variables. The between-subjects factor was stimulation (active vs. sham) and the within-subjects factor was time (T0 vs. T4). To deal with bias due to missing values, we used a linear mixed model repeated measures (LMMRM) analysis, and to adjust for possible intergroup differences at baseline, we included the respective baseline scores as covariates. The significance level was set at an alpha value of 0.05.

In addition, we calculated effect sizes based on Cohen’s d for group differences and time effects (both based on differences in LMMRM estimated means related to pooled observed standard deviations). As predefined in the study protocol, primary analyses were performed in the per-protocol (PP) sample (patients with at least 16 iTBS sessions; n = 40) and additionally in the intention-to-treat (ITT) sample (patients who were included and randomized, N = 53). All analyses were performed with IBM SPSS V29 [61].

## Results

Between January 2021 and August 2023, 282 patients were screened for eligibility, and 53 (18.8%) were eligible for inclusion. The flow of patients through the study is shown as a CONSORT flowchart in Fig. 1.

Of the 53 patients included, 25 (47.2%) were randomly allocated to the active iTBS group and 28 (52.8%) to the sham group. In the active group, 8 patients (32.0%) dropped out or were excluded from the analyses because of the pre-defined criterion (i.e., < 16 stimulation sessions) compared with 5 patients (17.4%) in the sham group (*p* = 0.23). Accordingly, a total of 40 patients were included in the PP analyses (active, n = 17 [42.5%]; sham group, n = 23 [57.5%]).

A comparison of the 40 patients in the PP population with the 13 patients who dropped out (see also Supplementary Table S2) revealed some differences in sex (percentage of female patients: 85.0% vs. 92.3%, respectively; *p* = 0.062), weight (mean [SD], 79.8 [21.3] kg vs 65.3 [10.5] kg, respectively; *p* = 0.002), and body mass index (BMI; mean [SD], 27.2 [6.2] vs. 23.7 [3.6], respectively; *p* = 0.018) but not in any other variables (i.e., age, symptom scale scores, time since first BPD diagnosis, and years of education).

The sample characteristics of the 40 patients included in the analyses (PP population) are shown for both treatment groups (ACTIVE-iTBS vs. SHAM) in Table 1. Mean age of patients was 25.2 years (SD = 6.2) and 85.0% (n = 34) were women. BPD was first diagnosed on average 1.7 years prior to study inclusion (SD = 3.8). BPD symptoms according to the BSL-23 were on average moderate (mean sum score = 42.0; SD = 16.4), and depressive symptoms according to the BDI-II were moderate to severe (mean = 33.3; SD = 10.9). All these characteristics were not significantly different between treatment groups as were self-assumed iTBS-treatment ('active', 'sham', 'unknown/unsure'; see Table 1), however some differences in psychotropic drug treatment were found between the groups: Whereas the percentage of patients treated with any antidepressant was similar in the two groups (60.9% in sham and 58.8% in active; *p* = 0.89), significantly more patients in the sham group received a selective serotonin reuptake inhibitor (39.1%) than patients in the active group (5.9%; *p* = 0.026) and less SSNRIs (8.7% vs. 35.2% respectively; *p* = 0.053). In addition, 4 patients in the active group (23.5%) received a second-generation antipsychotic (1 olanzapine, 3 quetiapine) but none in the sham group did (*p* = 0.026); nevertheless, there was no significant intergroup difference for treatment with any antipsychotic (*p* = 0.11). We tested the effect of differences in psychotropic drugs (antidepressants and antipsychotics) on the main treatment outcomes (BSL-23 and BDI-II) separately and found no significant differences (see Supplementary Tables S3a,b and S4a,b).

**Table 1:** Characteristics of patients included by treatment group

	Sham, n = 23	Active, n = 17	Total, N = 40	*p*^a^
Age, mean (SD), y	25.5 (6.6)	24.8 (5.9)	25.2 (6.2)	.71
Sex: female, n (%)	19 (82.6)	15 (88.2)	34 (85.0)	.86
Handedness: right, n (%)	18 (78.3)	14 (82.4)	32 (80.0)	.75
Smoking: “yes,” n (%)	14 (60.9)	9 (52.9)	23 (57.5)	.87
Years of education (school, university, or occupational training), mean (SD)	13.8 (2.4)	13.6 (2.4)	13.7 (2.4)	.81
Employed, n (%)	13 (56.5)	7 (41.2)	20 (50.0)	.34
Years since first BPD diagnosis, mean (SD)	1.5 (3.0)	2.0 (4.9)	1.7 (3.8)	.72
BSL-23 sum score, mean (SD)	43.6 (17.1)	39.9 (15.8)	42.0 (16.4)	.49
BDI, mean (SD)	33.9 (9.9)	32.5 (12.4)	33.3 (10.9)	.69
MADRS total score, mean (SD)	22.1 (5.8)	21.3 (5.6)	21.8 (5.7)	.67
MADRS item 10 (suicidal thoughts), mean (SD)	1.5 (1.0)	1.3 (0.8)	1.4 (0.9)	.54
SCS, mean (SD)	2.0 (0.7)	2.1 (0.5)	2.0 (0.6)	.85
GAF, mean (SD)	53.4 (13.1)	52.3 (13.3)	53 (13.0)	.79
Psychotropic drugs, n (%)				
None	9 (39.1)	4 (23.5)	13 (32.5)	.30
Any antidepressant	14 (60.9)	10 (58.8)	24 (60.0)	.89
SSRI	9 (39.1)	1 (5.9)	10 (25.0)	.026*
SSNRI	2 (8.7)	6 (35.3)	8 (20.0)	*.053*
Any antipsychotic	2 (8.7)	5 (29.4)	7 (17.5)	.11
Second-generation antipsychotic	0 (0)	4 (23.5)	4 (10.0)	.026*
Number of active or sham sessions, mean (SD)	18.0 (1.4)	18.5 (1.3)	18.2 (1.4)	.29
Resting motor threshold, mean (SD)	50.6 (11.9)	49.8 (11.0)	50.3 (11.4)	.83
Intermittent theta burst stimulation stimulus intensity, mean (SD)	40.3 (9.5)	39.6 (8.8)	40.0 (9.1)	.80
Self-assessment of treatment, n (%)				.23
Unknown or unsure	4 (17.4)	2 (11.8)	6 (15.0)	
Sham	10 (43.5)	4 (23.5)	14 (35.0)	
Active	9 (39.1)	11 (64.7)	20 (50.0)	

Active, intermittent theta burst stimulation; BDI, Beck’s Depression Inventory (second edition, BDI-II [57]); BPD, Borderline Personality Disorder; BSL-23, 23-item Borderline Symptom List [56]; GAF, Global Assessment of Functioning scale [58]; MADRS, Montgomery-Åsberg Depression Rating Scale [49]; SCS, Self-Compassion Scale (German short version, SCS-D; [59]); Sham, sham stimulation; SSNRI, selective serotonin noradrenalin reuptake inhibitor; SSRI, selective serotonin reuptake inhibitor

^a^Significance level for group differences: t test was used for continuous measures, Mann–Whitney test was used if normal distribution was not given (applies only to “Years since first Borderline Personality Disorder diagnosis”), Chi^2^ was used for frequencies/proportions, and exact testing was used in case of low cell frequencies

*p ≤ .05; italics values: .05 < *p* < .10

**Fig. 2:**
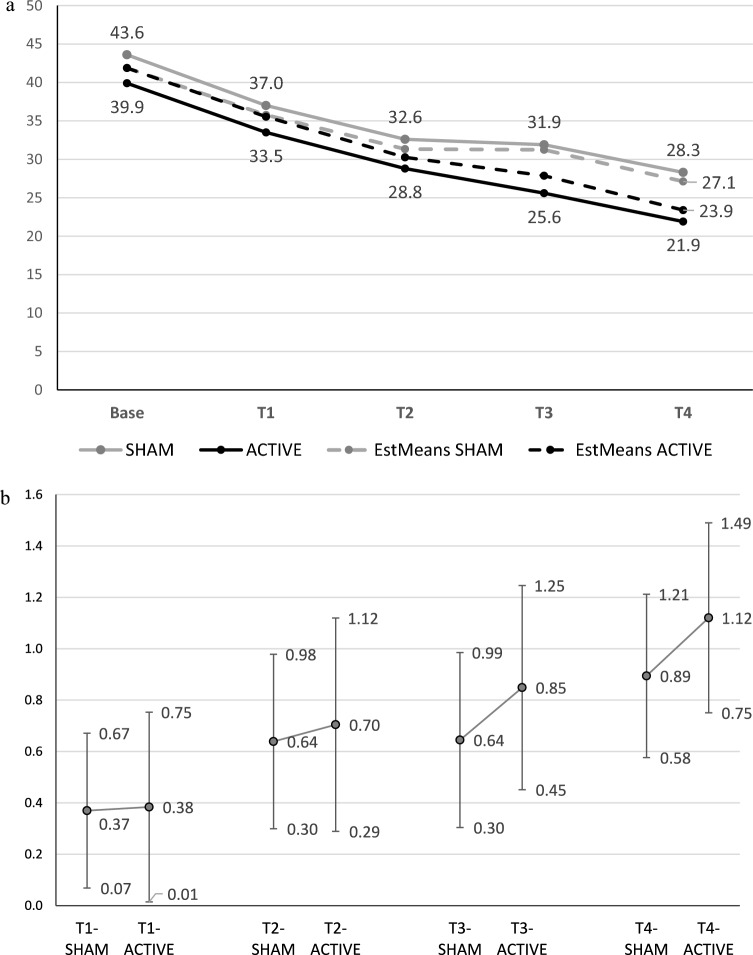
Results of the 23-item Borderline Symptom List. **a** Symptoms according to the 23-item Borderline Symptom List (BSL-23; observed values and estimated means from a mixed model repeated measures analysis) for active intermittent theta-burst stimulation (iTBS) and sham stimulation. Results of the mixed model analysis: BSL-23 baseline score, *p* < .001; group, *p* = .52; time, *p* < .001; and group*time: *p* = .89. Post hoc comparisons found no significant difference between active iTBS and sham stimulation at any time point. Active, intermittent theta-burst stimulation group; EstMeans, estimated means; Sham, sham stimulation group; T1, week 1; T2, week 2; T3, week 3; T4, week 4. **b** Effect sizes (95% confidence intervals) of change in BSL-23 from baseline to each time point for active iTBS and sham stimulation. Effect sizes according to Cohen’s d; estimated means from a mixed model repeated measures analysis related to pooled group standard deviation at baseline. *Active* intermittent theta-burst stimulation group, *Sham* sham stimulation group, *T1* week 1, *T2* week 2, *T3* week 3, *T4* week 4

The results of the LMMRM analysis for the primary outcome, BSL-23 after 4 weeks of active or sham stimulation, with the (BSL-)baseline score as a covariate are shown in Table 2 (including effect sizes for group differences). Additional parameters (beta estimates, standard errors, T-scores and 95%-CIs) are given in the supplement (Table S5). Figure 2a shows the course of the observed and estimated means from the LMMRM analysis for BSL-23. Detailed information on observed values (n, mean, SD) are given for the BSL-23 and the secondary outcomes in Supplementary Table S6. The LMMRM analysis found no significant difference between the sham and active groups over time (*p* = 0.89 for the interaction group*time). However, a highly significant effect for symptom reduction over time was found in both groups (*p* < 0.001 for the main effect *time*).

**Table 2:** Results of mixed model repeated measures analysis of the primary outcome 23-item Borderline Symptom List (BSL-23; including BSL-baseline score as covariate)

	Sham		Active				
Time, weeks	Estimated means	95% CI	Estimated means	95% CI	*p*^**a**^	Effect size^**b**^	95% CI
0	41.9^**c**^		41.9^**c**^				
1	35.8	30.8–40.7	35.5	29.4–41.6	.95	0.01	– 0.44 to 0.46
2	31.3	25.7–36.9	30.3	23.4–37.1	.81	0.06	– 0.42 to 0.54
3	31.2	25.6–36.9	27.9	21.3–34.4	.44	0.20	– 0.32 to 0.73
4	27.1	21.9–32.4	23.4	17.3–29.5	.36	0.23	– 0.26 to 0.72

Results of mixed model repeated measures analysis: 23-item Borderline Symptom List baseline score: *p* < .001; group, *p* = .52; time, *p* < .001; group*time, *p* = .89

^a^Post hoc significance level for group differences at specified times

^b^Effect size (Cohen's d) for group differences

^c^Constant score estimated by mixed model repeated measures analysis

The effect sizes (Cohen’s d) of the symptom reduction in BSL-23 from baseline to each time point are shown for each group in Fig. 2b. Overall, after 8 weeks’ DBT, an effect size (for symptom reduction) of 0.89 (Cohen’s d) was found for patients who received 4 weeks of add-on sham and of 1.12 (Cohen’s d) for patients who received 4 weeks of add-on active iTBS, which corresponds to an effect size of 0.23 for the group difference between sham vs. active (calculated from LMMRM estimates). Based on the F-value for the group*time effect an overall effect size of d = 0.15 was calculated.

**Fig. 3:**
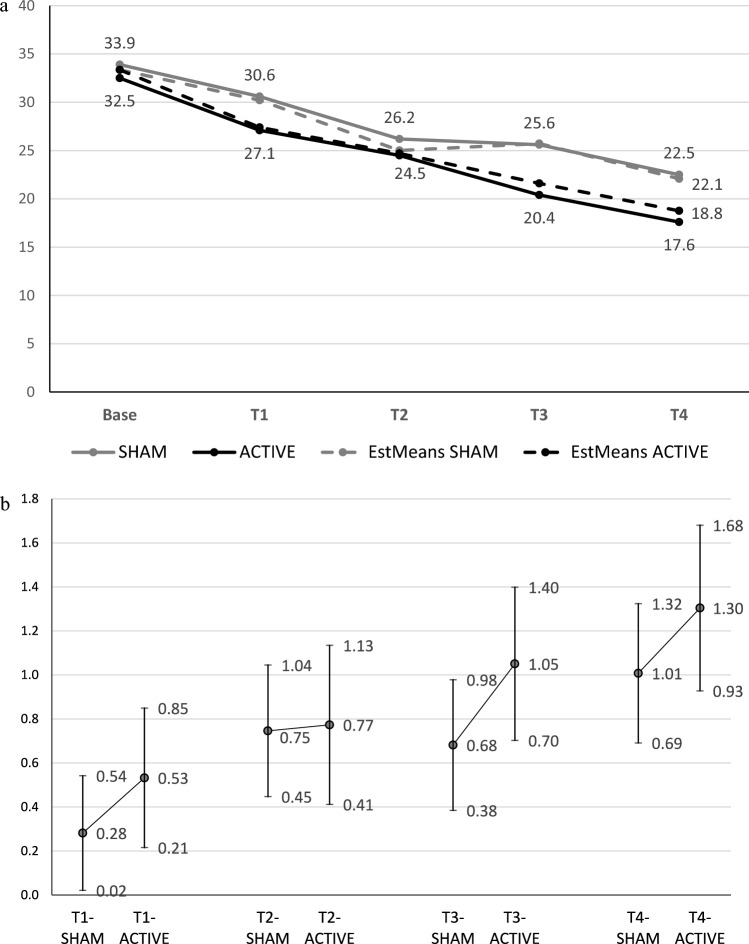
Results of the Beck Depression Inventory. **a** Symptoms according to the Beck Depression Inventory (BDI-II; observed values and estimated means from a mixed model repeated measures analysis) for active intermittent theta-burst stimulation (iTBS) and sham stimulation. Results of the mixed model analysis: BDI baseline score, *p* < .001; group, *p* = .22; time, *p* < .001; and group*time, *p* = .23. Post hoc comparisons found no significant difference between active and sham at any time point. **b** Effect sizes (95% confidence intervals) of change in BDI from baseline to each time point for active iTBS and sham. Effect sizes according to Cohen’s d; estimated means from a mixed model analysis related to pooled group standard deviation at baseline. *Active* intermittent theta-burst stimulation group, *Sham* sham stimulation group, *T1* week 1, *T2* week 2, *T3* week 3, *T4* week 4

The results of the LMMRM analyses regarding differences in course of secondary outcome measures between active and sham (including the respective baseline score as covariate if at least 3 measurements were scheduled) are shown for BDI scores in Table 3. Additional parameters (beta estimates, standard errors, T-scores and 95%-CIs) are given in the supplement (Table S7). Figure 3a, b shows the course of BDI over time (observed and estimated means) by treatment group and includes effect sizes (Cohen’s d) for symptom change (compared with baseline). No significant difference was found in the time course between groups (*p* = 0.23). Again, in both groups symptom reduction was highly significant (*p* < 0.001), with an overall effect size of 1.01 for sham and 1.30 for active iTBS.

**Table 3:** Results of mixed model repeated measures analysis of the secondary outcome Beck’s Depression Inventory (including BDI baseline score as a covariate)

	Sham		Active				
Time, weeks	Estimated means	95% CI	Estimated means	95% CI	*p*^a^	Effect size^b^	95% CI
0	33.4^c^		33.4^c^				
1	30.2	27.3–33.1	27.4	23.9–30.9	.22	0.27	– 0.17 to 0.71
2	25.0	21.7–28.4	24.7	20.7–28.7	.91	0.03	– 0.43 to 0.49
3	25.7	22.4–29.1	21.6	17.7–25.5	.11	0.37	– 0.09 to 0.83
4	22.1	18.5–25.6	18.8	14.5–23	.23	0.29	– 0.19 to 0.78

Results of mixed model repeated measures analysis: Beck’s Depression Inventory baseline score, *p* < .001; group, *p* = .22; time, *p* < .001; group*time, *p* = .23

^a^Post hoc significance level for group differences at specified times

^b^Effect size (Cohen’s d) for group differences

^c^Constant score estimated by mixed model repeated measures analysis

The results of the other secondary outcomes are summarized in Table 4. No significant group differences were found in any of the measures (MADRS, SCS, and GAF) for the interaction group*time, and DBT showed a highly favorable treatment effect (*p* < 0.01 for all main effects of *time*). Although not statistically significant, GAF effect sizes showed same advantages of active (d = 0.90) over sham stimulation, but SCS effect sizes were similar (1.07 vs. 1.09) and MADRS ones were lower (1.68 vs. 1.87).

**Table 4:** Results of mixed model repeated measures analysis of various secondary outcomes

	Sham		Active		
	Estimated means^a^ (95% CI)	Effect size^b^ (95% CI)	Estimated means^a^ (95% CI)	Effect size^b^ (95% CI)	*p*^c^
MADRS	11.0 (8.2–13.8)	1.87 (1.37–2.36)	12.1 (8.8–15.4)	1.68 (1.10–2.25)	.87
SCS	2.7 (2.4–3.0)	1.09 (1.53–0.66)	2.7 (2.4–3.1)	1.06 (1.57–0.56)	.93
GAF	61.0 (55.0–67.1)	0.58 (-0.03 to 1.18)	64.2 (57.2–71.2)	0.90 (0.20–1.60)	.48

Mixed model repeated measures analysis including Montgomery-Åsberg depression rating scale baseline score (3 measurements); SCS and GAF were assessed only twice (baseline and endpoint/T4); all main effects of time were (highly) significant (*p* < .01)

Active, intermittent theta burst stimulation; GAF, Global Assessment of Functioning scale [58]; MADRS, Montgomery-Åsberg depression rating scale [49]; SCS, Self-Compassion Scale (German short version, SCS-D; [59]); Sham, sham stimulation

^a^At endpoint/T4

^b^According to Cohen’s d for change to baseline score (difference in estimated means from mixed model repeated measures analysis related to pooled group SD at baseline)

^c^Significance level for group*time interaction

In addition to the PP analyses, we performed the same analyses in the ITT sample (all 53 patients included and randomized, active, n = 25; sham, n = 28). All the results for the primary (BSL-23) and secondary outcomes (BDI, MADRS, SCS, and GAF) were similar to those in the PP sample (i.e., no significant effects for time*group interaction, and highly significant effects for the main effect *time*); results are shown in Supplementary Table S8.

Results of further analyses of group differences in response and remission were also not significant (see Supplementary Table S9).

Regarding side effects or (serious) adverse events, we found no significant differences between treatment groups (see Table 5). Only one serious adverse event was observed: One patient in the active group attempted suicide and had to be transferred to a general hospital. The adverse event was rated as mild and not related to the trial.

**Table 5:** Adverse events in intention-to-treat sample (N = 53 patients included and randomized)

	Sham (n = 28)	Active (n = 25)	Total (N = 53)	*p*^a^
Patients with any AE	13 (46.4)	13 (52.0)	26 (49.1)	.69
Headache	9 (32.1)	6 (24.0)	15 (28.3)	.51
Total frequency of headaches (n)	16	6	22	
Other pain	1 (3.6)	1 (4.0)	2 (3.8)	.94
Nausea, vomiting	1 (3.6)	1 (4.0)	2 (3.8)	.94
Total frequency of nausea, vomiting (n)	1	2	3	
Malaise	0 (0)	1 (4.0)	1 (1.9)	.29
Dizziness	1 (3.6)	0 (0)	1 (1.9)	.94
Allergy	3 (10.7)	1 (4.0)	4 (7.5)	.36
Total frequency of allergy (n)	5	1	6	
Tearing and itching of the eye	0 (0)	2 (8.0)	2 (3.8)	.22
Total frequency of tearing/itching (n)	0	3	3	
Suicidal thoughts	1 (3.6)	0 (0)	1 (1.9)	.94
Dissociations	1 (3.6)	0 (0)	1 (1.9)	.94
COVID-19	0 (0)	2 (8)	2 (3.8)	.13
Other AEs (scheduled surgery, jaw twitching, neck complaints)	1 (3.6)	2 (8)	3 (5.7)	.47
Severity of AEs				.32
Mild	9 (69.2)	12 (92.3)	21 (80.8)	
Moderate	4 (30.8)	1 (7.7)	5 (19.2)	
Severity of AEs, mean (SD)	1.3 (0.5)	1.1 (0.3)	1.2 (0.4)	.15
Relatedness of AEs to treatment				.74
Probable	1 (7.7)	2 (15.4)	3 (11.5)	
Possible	7 (53.8)	7 (53.8)	14 (53.8)	
Unlikely	0 (0)	1 (7.7)	1 (3.8)	
Not related	5 (38.5)	3 (23.1)	8 (30.8)	
Relatedness of AEs, mean (SD)	2.7 (1.1)	2.4 (1)	2.5 (1.1)	.47

Given are the n (%) of patients with the respective adverse event (unless otherwise specified)

*AE* adverse event

^a^Significance level for group differences; t test was used for continuous measures, Mann–Whitney test if normal distribution was not given, Chi^2^ for frequencies / proportions, and exact testing in case of low cell frequencies

## Discussion

This study investigated whether iTBS as an add-on to DBT for BPD in an inpatient routine care setting improves the reduction of BPD-specific and depressive symptoms. In contrast to our hypothesis, we found no significant difference between the active and sham groups regarding overall BPD symptoms as assessed by the BSL-23. In addition, there were no significant differences in terms of depressive symptoms as measured by the BDI-II and MADRS or in more specific symptoms such as self-compassion (SCS).

Although we found no differences between the active and sham groups, both showed a highly significant improvement in BPD-specific and depressive symptoms over time. This finding is consistent with previous studies showing the efficacy of DBT in treating BPD [10, 13]. To date, few studies have evaluated iTBS as an add-on treatment to psychotherapy, and results have been mixed. A pilot study [44] found higher smoking cessation abstinence rates when iTBS was combined with CBT, although craving was not reduced [44]. Likewise, in panic disorder combined iTBS and CBT showed no significant differences in clinical improvement between the active and sham groups, possibly because of a ceiling effect [63]; a ceiling effect may also have been one reason why the current study found no significant difference between active and sham in BPD. The high effect sizes for overall improvement (sham, d = 0.89; active, d = 1.12) are notable and indicate that DBT alone is highly effective, making it more challenging to detect an additional effect of add-on iTBS. Surprisingly, iTBS was not more effective for comorbid depressive symptoms. Possible reasons could be the ceiling effect of psychotherapy discussed above, as well as differences in underlying pathology. Ceresa et al. [64] noted that BPD and MDD share some neurobiological underpinnings (e.g. changes in the amygdala). However, the depression in BPD may differ from typical melancholic depression and may therefore be more prone to anger, dysphoria or even hostility [64]. Furthermore, we applied stimulation only to the left DLPFC, which is associated with emotion regulation and impulsivity and is one of the most frequently stimulated areas in psychiatric treatment [35]. However, although emotion regulation is commonly associated with fronto-limbic imbalance in BPD, it remains unclear whether the left DLPFC is the optimal target region for stimulation [20].

Although the non-significant results indicate that iTBS is not sufficiently effective as an add-on to DBT, the results showed a distinct tendency for some benefit in the active group, as indicated by increasing differences in effect sizes for group differences of up to 0.23 (BSL-23) and 0.29 (BDI-II) at four weeks (see Figs. 2b, 3b). Because this was a pilot study with a rather small sample size, we performed a post hoc power analysis with the effect size obtained in the study (d = 0.23) and found that a sample size of N = 106 would be required to reach significance (alpha = 0.05; power = 0.80). Even though the effect size of d = 0.23 (for group differences) is rather small and has questionable clinical significance, the observed trend may indicate that the group difference would be larger if iTBS was applied for more than four weeks. Nevertheless, some patients with BPD may also benefit from a small effect of iTBS as an add-on to DBT given the therapeutic and general health care challenges in BPD treatment. Accordingly, our pilot trial indicates the feasibility of iTBS add-on to DBT and the need for future studies with sufficient power and sample size to further investigate the additional effect of iTBS, whether a significant difference becomes apparent after a longer observation period, and whether the positive effects of the combined treatment are more persistent than those of DBT alone. As an additional point, studies show variation in the optimal duration of rTMS treatment for depression [65], so the optimum duration of rTMS/iTBS remains unclear.

We found a similar non-significant trend in self-reported depressive symptoms (BDI-II), but not for clinician-rated depressive symptoms (MADRS). Differences between self-reported and clinician ratings of depression are common in the literature [66]. Reasons for this discrepancy include biases in symptom severity and acquiescence bias in self-reported measures and biases related to expectations of treatment efficacy in clinician ratings [66]. On the other hand, we found a positive trend towards superiority of add-on active iTBS also in clinician-rated general functioning (GAF; Cohen’s d for group difference after 4 weeks, 0.32), whereas self-assessed self-compassion (SCS) was similar in both groups (d = 0.03). Nevertheless, the trends should not be overestimated because the effects were not significant. Therefore, we recommend that future large multi-center, prospective studies evaluate add-on iTBS to DBT.

Our finding of a significant effect of time confirmed that 8 weeks of DBT is effective in treating BPD symptoms in an inpatient treatment setting. These results confirm earlier findings of our study group that 8-week DBT is highly effective and comparable to 12-week DBT. The 12-week program is more commonly used in inpatient treatment, although there is considerable variability in treatment durations [15, 68, 69]. This finding aligns with other studies [e.g. 68, 69], which showed that shorter DBT could be equally beneficial. Taken together, results suggest that DBT can be shortened, making treatment more efficient.

In our study, the effect size of DBT was quite large compared with the various results in the literature [67, 69, 70]. The high baseline symptom scores in our study (e.g., the mean BDI in the two groups was 32.5 and 33.9, corresponding to severe depression) may have contributed to the favorable effect size. It should also be noted that the patients had already been undergoing inpatient DBT for 4 weeks when they started the study.

### Limitations

Our single-blind randomized controlled trial also has some limitations. First, because this was a pilot study the sample size may have been too small to detect a significant effect. Also, the trial was not double blind because such a design was not feasible in our routine care setting. Nevertheless, we tried to address this topic by performing blinded data analyses and checking whether patients were able to guess their treatment group. Furthermore, we had strict inclusion and exclusion criteria, which may have led to a selection bias and may limit external validity. Also, the study had no follow-up, so we cannot draw any conclusions about the sustainability of the effects. In addition, we focused on emotion dysregulation as a major symptom of BPD and therefore stimulated the left DLPFC, but future studies could also focus on other brain areas (e.g. the right DLPFC [71]). Furthermore, different stimulation protocols could be used like conventional rTMS or cTBS [71, 72]. The iTBS took place on the same ward as routine psychiatric treatment, which might have had a favorable effect on patient compliance and feasibility in general. In addition, we only applied the stimulation after the patients had already completed 4 weeks of DBT, so future studies should further investigate the timing of iTBS as an add-on to DBT.

## Conclusion

We found no significant difference between active iTBS and sham stimulation as an add-on to DBT. However, the study showed a distinct trend in favor of active iTBS. Furthermore, the sample size of our pilot trial was rather small. Nevertheless, we showed that 8 weeks of DBT was effective in reducing BPD-specific and depressive symptoms, a finding that may contribute to improved care for BPD patients. Future studies should use larger sample sizes and multi-center prospective designs. Furthermore, iTBS should be studied as an add-on to other specialized psychotherapeutic interventions because it is quick and easy to apply.

## Data availability:

Data will be provided to any researcher by the corresponding author upon reasonable request.

## Supplementary Information

Below is the link to the electronic supplementary material.

Supplementary file1 (DOCX 52 KB)
