# Supplementary material for: Effects of intermittent theta burst stimulation add-on to dialectical behavioral therapy in borderline personality disorder: results of a randomized, sham-controlled pilot trial
Source: Eur Arch Psychiatry Clin Neurosci. 2024 Sep 19;275(8):2301–14. doi: 10.1007/s00406-024-01901-0 (PMC12638378; doi:10.1007/s00406-024-01901-0)
Supplement: Supplementary file 1 — Supplementary file1 (DOCX 52 KB) [file 406_2024_1901_MOESM1_ESM.docx]

**Supplementary data**

**Effects of intermittent theta burst stimulation add-on to dialectical behavioral therapy in borderline personality disorder: Results of a randomized, sham-controlled pilot trial**

Milenko Kujovic^a^, Christian Bahr^a^, Mathias Riesbeck^a^, Daniel Benz^a^, Lena Wingerter^a^, Martina Deiß^a^, Zsofia Margittai^a^, Dirk Reinermann^a^, Christian Plewnia^b^, Eva Meisenzahl^a^

^a^ Department of Psychiatry and Psychotherapy, Medical Faculty and University Hospital Düsseldorf, Heinrich-Heine-University Düsseldorf, Düsseldorf, Germany

^b^ Department of Psychiatry and Psychotherapy, University Hospital of Psychiatry and Psychotherapy, Tübingen, Germany

**Corresponding author:** Milenko.Kujovic@lvr.de

Table S1. Screening, baseline, and treatment-phase assessments of primary and secondary outcomes

For a more detailed description, see Kujovic et al. [45].

|  | **Screening** | **Baseline** | **Treatment phase Post** | | | |
| --- | --- | --- | --- | --- | --- | --- |
| **Assessment** | T0 | | T1  (end week 1) | T2  (end week 2) | T3  (end week 3) | T4  (end week 4) |
| Informed Consent | x |  |  |  |  |  |
| Inclusion/exclusion criteria | x |  |  |  |  |  |
| SCID-V-PD | x |  |  |  |  |  |
| Mini-DIPS OA | x |  |  |  |  |  |
| MADRS | x |  |  | x |  | x |
| BSL-23 |  | x | x | x | x | x |
| BDI-II |  | x | x | x | x | x |
| Resting motor threshold |  | x |  | x |  |  |

BDI, Beck’s Depression Inventory (second edition, BDI-II; [58]); BSL-23, 23-item Borderline Symptom List ([57]); GAF, Global Assessment of Functioning scale [59]; MADRS, Montgomery-Åsberg Depression Rating Scale [50]; Mini-DIPS OA, Diagnostic Interview for Mental Disorders [48]; SCID-V-PD, Structured Clinical Interview for DSM-5 Personality Disorders [49]

Table S2: Characteristics of patients who dropped out or were excluded (because they received < 16 stimulation sessions) vs. patients who were included

|  | **Drop-outs/Excluded patients (n = 13)** | **Included patients (n = 40)** | **Total (N = 53)** | ***p*^1^** |
| --- | --- | --- | --- | --- |
| Age, mean (SD), y | 25.3 (5.9) | 25.2 (6.2) | 25.2 (6.1) | .96 |
| Sex: female n (%) | 12 (92.3) | 34 (85.0) | 46 (86.8) | .062 |
| Height, mean (SD), cm | 166.2 (7.7) | 169.9 (7.4) | 169 (7.6) | .13 |
| Weight, mean (SD), kg | 65.3 (10.5) | 79.8 (21.3) | 76.3 (20.1) | **.002**** |
| BMI, mean (SD) | 23.7 (3.6) | 27.2 (6.2) | 26.3 (5.8) | **.018*** |
| Education (school, university or job) mean (SD), y | 13.6 (2.5) | 13.7 (2.4) | 13.7 (2.4) | .85 |
| MADRS total score, mean (SD) | 22.3 (8.6) | 21.8 (5.7) | 21.9 (6.4) | .83 |
| MADRS item 10 (suicidal thoughts), mean (SD) | 1.8 (1.0) | 1.4 (0.9) | 1.5 (1.0) | .23 |
| BSL-23, mean (SD) | 39.3 (20.3) | 42 (16.4) | 41.4 (17.2) | .63 |
| BDI, mean (SD) | 29.5 (13.7) | 33.3 (10.9) | 32.4 (11.6) | .33 |
| SCS, mean (SD) | 2.1 (0.8) | 2 (0.6) | 2.1 (0.6) | .64 |
| GAF mean (SD) | 51.7 (6.7) | 53.0 (13) | 52.7 (11.8) | .75 |
| Years since first BPD diagnosis, mean (SD) | 1.5 (2.1) | 1.7 (3.8) | 1.7 (3.5) | .53 |
| Randomized treatment, n (%) - Sham - Active | 5 (38.5) 8 (61.5) | 23 (57.5) 17 (42.5) | 28 (52.8) 25 (47.2) | .23 |

BDI, Beck’s Depression Inventory (second edition, BDI-II; [58]); BMI, body mass index; BPD, Borderline Personality Disorder; BSL-23, 23-item Borderline Symptom List [57]; GAF, Global Assessment of Functioning scale [59]; MADRS, Montgomery-Åsberg Depression Rating Scale [50]; SCS, Self-Compassion Scale (German short version, SCS-D; [60])

**^1^** Significance level for group differences; t test was used for continuous measures, Mann-Whitney test was used if normal distribution was not given (applies only to “Years since first BPD diagnosis”), and Chi^2^ was used for frequencies / proportions

Table S3a: Results of mixed model repeated measures analysis comparing effect of antidepressant treatment (AD) on the Borderline Symptom List score (including baseline score as covariate, time points 1-4 as repeated factor, AD and AD*time interaction)

| **Antidepressant treatment** | **BSL-23 endpoint estimated means** | **Standard error** | **df** | **95% CI** |
| --- | --- | --- | --- | --- |
| SSRI | 22.6 | 4.0 | 41.2 | 14.5 - 30.7 |
| SSNRI | 28.3 | 4.5 | 40.6 | 19.3 - 37.3 |
| Other | 25.9 | 2.7 | 40.6 | 20.4 - 31.3 |

Results of mixed model repeated measures analysis: Borderline Symptom List baseline score, *p* < .001; antidepressant treatment, *p* = .38; time, *p* < .001; antidepressant treatment*time: *p* = .99

BSL-23, 23-item Borderline Symptom List [56]; SSNRI, selective serotonin noradrenalin reuptake inhibitor; SSRI, selective serotonin reuptake inhibitor

Table S3b: Results of mixed model repeated measures analysis comparing effect of antidepressant treatment (AD) on Beck’s Depression Inventory score (including baseline score as covariate, time points 1-4 as repeated factor, AD, and AD * time interaction)

| **Antidepressant treatment** | **BDI endpoint estimated means** | **Standard error** | **df** | **95% CI** |
| --- | --- | --- | --- | --- |
| SSRI | 19.7 | 2.7 | 41.0 | 14.3 - 25.2 |
| SSNRI | 24.4 | 3.2 | 43.0 | 18.0 - 30.7 |
| Other | 19.8 | 1.8 | 40.6 | 16.2 - 23.5 |

Results of mixed model repeated measures analysis: Beck’s Depression Inventory baseline score, *p* < .001; AD, *p* = .16; time, *p* < .001; antidepressant treatment*time, *p* = .31
BDI, Beck’s Depression Inventory [58]; SSNRI, selective serotonin noradrenalin reuptake inhibitor; SSRI, selective serotonin reuptake inhibitor

Table S4a: Results of mixed model repeated measures analysis comparing effect of antipsychotic treatment (AP) on 23-item Borderline Symptom List score (including baseline score as covariate, time points 1-4 as repeated factor, AP, and AP*time interaction)

| **Antipsychotic treatment** | **BSL-23 endpoint estimated means** | **Standard error** | **df** | **95% CI** |
| --- | --- | --- | --- | --- |
| SGA | 19.5 | 5.5 | 41.5 | 8.3 - 30.6 |
| None / Other | 26.4 | 2.1 | 41.4 | 22.2 - 30.6 |

Results of mixed model repeated measures analysis: BSL-23 baseline score, *p* < .001; AP, *p* = .35; time, *p* = .012; AP*time, *p* = .94
BSL-23, 23-item Borderline Symptom List [57]; SGA, second-generation antipsychotic

Table S4b: Results of mixed model repeated measures analysis comparing effect of antipsychotic treatment (AP) on Beck’s Depression Inventory score (including baseline score as covariate, time points 1-4 as repeated factor, AP, and AP*time interaction)

| **Antipsychotic treatment** | **BDI endpoint estimated means** | **Standard error** | **df** | **95% CI** |
| --- | --- | --- | --- | --- |
| SGA | 19.0 | 3.8 | 41.8 | 11.3 - 26.7 |
| None / Other | 21.0 | 1.5 | 42.2 | 18.0 - 23.9 |

Results of mixed model repeated measures analysis: Beck’s Depression Inventory baseline score, *p* < .001; antipsychotic, *p* = .41; time: *p* = .01; AP*time, *p* = .21
BDI, Beck’s Depression Inventory [58]; SGA, second-generation antipsychotic

Table S5: BSL-23: parameter estimates of mixed model analysis

|  | **Estimate (beta)** | **Standard error** | **df** | **T** | **p** | **95%-CI** |
| --- | --- | --- | --- | --- | --- | --- |
| Intercept | -7.04 | 4.80 | 50.21 | -1.47 | 0.15 | -16.68 / 2.60 |
| Covariate (baseline) | 0.73 | 0.09 | 42.43 | 7.76 | <.001 | 0.54 / 0.92 |
| Group (0 / sham) | 3.73 | 3.99 | 41.50 | 0.93 | 0.36 | -4.33 / 11.79 |
| Group (1 / active) | 0 ^1)^ | 0 | - | - | - | - |
| Time (1) | 12.15 | 3.73 | 87.52 | 3.26 | 0.002 | 4.75 / 19.56 |
| Time (2) | 6.87 | 3.61 | 82.71 | 1.90 | 0.061 | -0.31 / 14.04 |
| Time (3) | 4.48 | 2.73 | 62.61 | 1.64 | 0.11 | -0.98 / 9.94 |
| Time (4) | 0 ^1)^ | 0 | - | - | - | - |
| Time (1) * Group (0) | -3.50 | 4.85 | 87.47 | -0.72 | 0.47 | -13.13 / 6.13 |
| Time (2) * Group (0) | -2.65 | 4.67 | 83.42 | -0.57 | 0.57 | -11.94 / 6.63 |
| Time (3) * Group (0) | -0.36 | 3.60 | 62.64 | -0.10 | 0.92 | -7.55 / 6.82 |
| Time (4) * Group (0) | 0 ^1)^ | 0 | - | - | - | - |
| Time (1) * Group (1) | 0 ^1)^ | 0 | - | - | - | - |
| Time (2) * Group (1) | 0 ^1)^ | 0 | - | - | - | - |
| Time (3) * Group (1) | 0 ^1)^ | 0 | - | - | - | - |
| Time (4) * Group (1) | 0 ^1)^ | 0 | - | - | - | - |

^1)^ The parameter is set to '0' by the model procedure due to redundancies

Table S6: Treatment course of observed values in primary (23-item Borderline Symptom List) and secondary outcome measures by treatment group

|  | **Sham** | | | **Active** | | |
| --- | --- | --- | --- | --- | --- | --- |
| **Outcome by time** | **n** | **Mean** | **SD** | **n** | **Mean** | **SD** |
| BSL-23 |  |  |  |  |  |  |
| 0 | 23 | 43.6 | 17.1 | 17 | 39.9 | 15.8 |
| 1 | 23 | 37.0 | 17.8 | 15 | 33.5 | 17.1 |
| 2 | 21 | 32.6 | 19.6 | 13 | 28.8 | 17.1 |
| 3 | 22 | 31.9 | 17.7 | 16 | 25.6 | 15.3 |
| 4 | 23 | 28.3 | 17.5 | 17 | 21.9 | 15.3 |
| BDI-II |  |  |  |  |  |  |
| 0 | 23 | 33.9 | 9.9 | 17 | 32.5 | 12.4 |
| 1 | 23 | 30.6 | 10.9 | 15 | 27.1 | 10.1 |
| 2 | 21 | 26.2 | 11.3 | 13 | 24.5 | 11.5 |
| 3 | 22 | 25.6 | 11.1 | 16 | 20.4 | 11.3 |
| 4 | 23 | 22.5 | 11.9 | 16 | 17.6 | 10.8 |
| SCS |  |  |  |  |  |  |
| 0 | 23 | 2.0 | 0.7 | 17 | 2.1 | 0.5 |
| 4 | 23 | 2.7 | 0.7 | 17 | 2.7 | 0.7 |
| MADRS |  |  |  |  |  |  |
| 0 | 23 | 22.1 | 5.8 | 17 | 21.3 | 5.6 |
| 2 | 19 | 13.9 | 6.6 | 15 | 14.3 | 5.1 |
| 4 | 23 | 11.2 | 6.7 | 17 | 11.9 | 6.7 |
| GAF |  |  |  |  |  |  |
| 0 | 23 | 53.4 | 13.1 | 17 | 52.3 | 13.3 |
| 4 | 23 | 61.0 | 16.5 | 17 | 64.2 | 10.3 |

Active, intermittent theta burst stimulation; BDI, Beck’s Depression Inventory (second edition, BDI-II; [58]); BSL-23, 23-item Borderline Symptom List [57]; GAF, Global Assessment of Functioning scale [59]; MADRS, Montgomery–Åsberg depression rating scale [50]; SCS, Self-Compassion Scale (German short version, SCS-D; [60]); Sham, sham stimulation

Table S7: BDI: parameter estimates of mixed model analysis

|  | **Estimate (beta)** | **Standard error** | **df** | **T** | **p** | **95%-CI** |
| --- | --- | --- | --- | --- | --- | --- |
| Intercept | -5.48 | 3.63 | 47.93 | -1.51 | 0.14 | -12.77 / 1.81 |
| Covariate (baseline) | 0.73 | 0.09 | 39.27 | 7.89 | <.001 | 0.54 / 0.91 |
| Group (0 / sham) | 3.32 | 2.73 | 42.12 | 1.22 | 0.23 | -2.19 / 8.83 |
| Group (1 / active) | 0 ^1)^ | 0 | - | - | - | - |
| Time (1) | 8.64 | 2.20 | 88.33 | 3.92 | <.001 | 4.26 / 13.02 |
| Time (2) | 5.95 | 2.08 | 87.95 | 2.85 | 0.005 | 1.8 / 10.09 |
| Time (3) | 2.84 | 1.51 | 59.19 | 1.88 | 0.07 | -0.19 / 5.87 |
| Time (4) | 0 ^1)^ | 0 | - | - | - | - |
| Time (1) * Group (0) | -0.51 | 2.85 | 87.37 | -0.18 | 0.86 | -6.17 / 5.15 |
| Time (2) * Group (0) | -3.02 | 2.67 | 87.16 | -1.13 | 0.26 | -8.33 / 2.30 |
| Time (3) * Group (0) | 0.81 | 1.98 | 59.52 | 0.41 | 0.68 | -3.16 / 4.78 |
| Time (4) * Group (0) | 0 ^1)^ | 0 | - | - | - | - |
| Time (1) * Group (1) | 0 ^1)^ | 0 | - | - | - | - |
| Time (2) * Group (1) | 0 ^1)^ | 0 | - | - | - | - |
| Time (3) * Group (1) | 0 ^1)^ | 0 | - | - | - | - |
| Time (4) * Group (1) | 0 ^1)^ | 0 | - | - | - | - |

^1)^ The parameter is set to '0' by the model procedure due to redundancies

Table S8: Results of mixed model repeated measures analyses of the primary and all secondary outcomes in the intention-to-treat population (N = 53 patients included and randomized; intermittent theta burst stimulation, n = 25; sham, n = 28)

|  | Sham | | Active | |  | |  |
| --- | --- | --- | --- | --- | --- | --- | --- |
|  | Estimated means**^1^**  (95% CI) | Effect size**^2^**  (95% CI) | Estimated means**^1^**  (95% CI) | Effect size**^2^**  (95% CI) | *p* Time*group**^3^** | | *p* Time**^4^** |
| BSL-23 | 26.7  (21.5 - 31.8) | 0.85  (0.54 - 1.16) | 22.8  (17.0 - 28.5) | 1.09  (0.74 - 1.44) | .87 | < .001 | |
| BDI | 21.5  (18.0 - 25.0) | 0.98  (0.45 - 0.88) | 18.8  (14.8 - 22.8) | 1.22  (0.59 - 1.07) | .41 | < .001 | |
| MADRS | 11.0  (8.2 - 13.9) | 1.80  (1.29 - 2.3) | 11.8  (8.6 - 15.1) | 1.65  (1.09 - 2.22) | .70 | .01 | |
| SCS | 2.7  (2.4 - 3.0) | 1.10  (0.61 - 1.59) | 2.8  (2.4 - 3.1) | 1.02  (0.47 - 1.56) | .79 | < .001 | |
| GAF | 61.0  (55.1 - 67.0) | 0.59  (0.14 - 1.04) | 64.5  (57.8 - 71.2) | 0.90  (0.40 - 1.41) | .45 | < .001 | |

Mixed model repeated measures analysis including baseline score on the 23-item Borderline Symptom List, Beck’s Depression Inventory (5 measurements), and Montgomery-Åsberg Depression Rating Scale (3 measurements); the Self-Compassion Scale, and Global Assessment of Functioning were assessed only twice (at baseline and endpoint/T4)

Active, intermittent theta burst stimulation; BDI, Beck’s Depression Inventory (second edition, BDI-II; [58]); BSL-23, 23-item Borderline Symptom List [57]; GAF, Global Assessment of Functioning scale [59]; MADRS, Montgomery-Åsberg Depression Rating Scale[50]; SCS, Self-Compassion Scale (German short version, SCS-D; [60]); Sham, sham stimulation

**^1^** At endpoint/T4

**^2^** According to Cohen’s d for change from baseline score (difference in estimated means from mixed model repeated measures analysis related to pooled group SD at baseline)

**^3^** Significance level for group*time interaction

**^4^** Significance level for main effect time

Table S9: Borderline Personality Disorder (23-item Borderline Symptom List) and depression (Beck’s Depression Inventory) symptom response and remission in patients with sham stimulation or intermittent theta burst stimulation

|  | **Sham (n = 23)** | **Active (n = 17)** | ***p*^1^** |
| --- | --- | --- | --- |
| **BSL-23** |  |  |  |
| % symptom reduction from baseline to T4, mean (SD) | 37.0 (28.3) | 43.8 (39.0) | .53 |
| Patients with … |  |  | .19 |
| - at least 50% reduction, n (%) | 6 (26.1) | 9 (52.9) |  |
| - at least 25%-49.9% reduction, n (%) | 11 (47.8) | 4 (23.5) |  |
| - less than 25% reduction, n (%) | 6 (26.1) | 4 (23.5) |  |
| Patients with symptom remission,**^2^** n (%) | 9 (39.1) | 11 (64.7) | .11 |
| **BDI** |  |  |  |
| % symptom reduction from baseline to T4, mean (SD) | 36.5 (27.7) | 44.6 (31.8) | .40 |
| Patients with … |  |  | .19 |
| - at least 50% reduction, n (%) | 9 (39.1) | 9 (56.3) |  |
| - at least 25%-49.9% reduction, n (%) | 7 (30.4) | 2 (12.5) |  |
| - less than 25% reduction, n (%) | 7 (30.4) | 5 (31.3) |  |
| Patients with symptom remission,**^3^** n (%) | 5 (21.7) | 7 (43.8) | .17 |

Active, intermittent theta burst stimulation; BSL-23, 23-item Borderline Symptom List [60]; BDI, Beck’s Depression Inventory (second edition, BDI-II; [58]); Sham, sham stimulation

**^1^** Significance level for group differences; t test was used for continuous measures, Chi^2^ for frequencies / proportions, and exact testing for low cell frequencies

**^2^** 23-item Borderline Symptom List: sum score of 25 or less according to Kleindienst et al. [S1]

**^3^** Beck’s Depression Inventory: sum score of 13 or less

**References (Supplement only)**

[S1] Kleindienst N, Jungkunz M, Bohus M. A proposed severity classification of borderline symptoms using the borderline symptom list (BSL-23). Borderline Personality Disorder and Emotion Dysregulation. 2020 Jun 1;7(1)
